# How can non-inferiority studies with mortality end points be ethically justified?

**DOI:** 10.1136/jme-2024-110517

**Published:** 2025-08-26

**Authors:** Dick Willems, Hanno Tan, Nikolaos Dagres, Marieke A R Bak

**Affiliations:** 1 Department of Ethics, Law and Humanities, Amsterdam UMC, University of Amsterdam, Amsterdam, The Netherlands; 2 Department of Clinical and Experimental Cardiology, Amsterdam UMC, University of Amsterdam, Amsterdam, The Netherlands; 3 Deutsches Herzzentrum der Charité, Berlin, Germany; 4 Ethics, Law and Humanities, Amsterdam UMC - Locatie AMC, Amsterdam, The Netherlands

**Keywords:** biostatistics, clinical trial, ethics, research, ethics, ethics committees, research

## Abstract

**Background:**

Non-inferiority (NI) studies with mortality end points are increasingly frequently conducted. They aim to show that a new treatment strategy does not entail an unacceptably higher mortality than the comparator. They raise specific ethical issues related to the rationale of the study, the NI margin, certainty and informed consent. There is a need for ethical reflection.

**Method:**

Analysis of ethical issues informed by a literature search using terms related to NI, mortality and ethics, in PubMed, CINAHL and Embase. Results are illustrated using the example of the PROFID-EHRA NI trial that the authors are involved in.

**Results:**

Justifications for conducting an NI study instead of a superiority study are often insufficient. The NI margin is most often taken from previous studies without additional justification. There is no consensus about how patients should be involved in the design and justification of the studies and about how participants should be informed.

**Discussion:**

We conclude that NI studies with mortality end points can be ethically justified if secondary benefits are proven and large enough for participants, and if the NI margin is acceptable to patients and ethics committees. Acceptability of the NI margin should be determined on a case-by-case basis and risks should be framed appropriately. The justification for choosing an NI rather than a superiority design should be made more explicitly. Further studies are needed on patients’ views about NI trials with mortality as an end point; also, the degree of certainty and the very distinction between primary and secondary outcomes deserve systematic study.

## Introduction

 Non-inferiority (NI) studies are designed to demonstrate that a new treatment or strategy is not less effective than another, usually the standard of care.^1^ NI studies, also those with mortality end points, are conducted increasingly frequently,^2^ and are often motivated by three aims that are usually combined. First, NI trials are conducted to reduce invasiveness, burden or side effects, while maintaining effectiveness. Demonstrating NI is necessary when a new treatment is not expected to be more effective, but offers advantages in other areas, such as side effects, ease of use or quality of life. Second, NI trials may be done to lower costs and increase global access, by comparing an existing treatment strategy with no treatment or with a cheaper or more accessible strategy. For instance, studies about treating uncomplicated sinusitis with or without antibiotics.^3^ Such studies may be justified on grounds of equity, especially when populations in lower income countries are involved. Third, NI trials are sometimes considered where head-to-head superiority trials comparing a new treatment strategy with the standard are expected to be too failure prone. NI trials that are purely based on this rationale offer no clinical benefit to participating patients, whose interest lies in better—not merely ‘no worse’—treatments. Such trials are often driven primarily by commercial interests, and the lack of clinical benefit makes them ethically unjustifiable.^4^

There is consensus that, for statistical reasons, NI cannot be interpreted as zero difference, because demonstrating this would require an enormous number of participants to be enrolled. Therefore, the protocol of NI trials usually specifies a threshold value for delta (ie, the difference) below which the new treatment is considered non-inferior (the NI margin). A small difference in effectiveness is often considered clinically acceptable by the medical community, and is therefore regarded as compatible with NI. In fact, NI trials are not what they say they are: instead of NI studies, they should be called ‘acceptable-inferiority trials’. But since ‘acceptable’ is a value judgement, this obviously raises the question: acceptable to whom? For the researchers, the physicians or the participants? We are aware that terms like this (other examples include ‘small’, ‘negligible’, ‘slight’, ‘not worse’), while unavoidable when discussing NI trials, are implicitly or explicitly value-laden. Because of the inevitability of accepting an NI margin, Garattini and Bertele,^4^ in a controversial *Lancet* paper, stated that NI trials are always unethical, because they require accepting some harm to participants without the benefit of a better primary outcome. As was pointed out in the discussion that followed,^5^ these authors disregard the trade-off character of NI studies: trading a potential, and minimal, loss of efficacy for other benefits such as fewer adverse effects, better compliance and lower costs.

While NI studies most often concern non-lethal diseases, they sometimes compare curative or preventative strategies for a lethal disease and, accordingly, have mortality or survival as their primary end point. These studies raise harder ethical questions than studies concerning non-lethal diseases, as has been noted by the European Medicines Evaluation Agency (EMEA) in a 2005 report, which stated that it is “very difficult to justify a non-inferiority margin of any size in a study where the treatment under consideration is used for the prevention of death or irreversible morbidity and there is no second chance for treatment. Discussion of the number of extra deaths that are acceptable is ethically very difficult”.^6^

The authors of this paper are involved in the ethics work package of the PROFID-EHRA trial (https://profid-project.eu/profid-ehra-trial/), a multinational NI study that compares two strategies to prevent sudden cardiac death (SCD) in patients with significant heart failure after they survived a myocardial infarction (MI): implantation versus non-implantation of an implantable cardioverter defibrillator (ICD); mortality is its primary end point.^7^

The PROFID-EHRA trial is based on the fact that some patients who are in the chronic phase after an MI are at increased risk of SCD. To prevent this, implantation of an ICD is the current standard of care. Current clinical guidelines advise prophylactic ICD implantation when the left ventricular ejection fraction (LVEF), a measure for heart failure, is reduced to <35% of the normal value. However, there is a situation of clinical equipoise, which is the basis for the justification of any clinical trial: first, the ICDs typically deliver appropriate therapy (an ICD shock) in only a minority of the patients who carry them, since lethal cardiac arrhythmias will not occur in most patients. Thus, most patients are currently exposed to the potentially severe complications due to ICD implantation without benefiting from the ICD. Second, the risk-benefit ratio of ICD implantation for primary prevention of SCD in patients with reduced LVEF has substantially changed during recent years because better drug treatment of post-MI patients has reduced SCD risk, while the complication rates associated with ICD implantation have remained unaltered. PROFID-EHRA is an investigator-driven clinical NI trial that plans to randomise 3595 post-MI patients who would receive an ICD according to current clinical guidelines. It aims to demonstrate that optimal medical therapy (OMT) without ICD implantation (index group) is not inferior to OMT with ICD implantation (control group) with respect to all-cause mortality within about 2.5 years of observation. The ethicists in the project were asked for advice about the ethical issues related to this study.

In this paper, we describe those ethical issues because they have relevance beyond the PROFID-EHRA trial. Clearly, NI studies are subject to the Declaration of Helsinki concerning the ethics of medical research with human subjects. However, they do raise specific ethical issues,^8 9^ related to, first, the rationale for doing an NI instead of a superiority study; second, the extent of inferiority that will be considered acceptable (the ‘NI margin’); third, the degree of certainty needed, and fourth, the information needed for informed patient consent. All four issues are discussed in the narrative review below, on the basis of a systematic literature search on the ethics of NI studies with mortality as end point (see box 1) and with references to the PROFID-EHRA trial as illustration. For a precise description of the search strategy and the Preferred Reporting Items for Systematic Reviews and Meta-Analyses flow chart, see online supplemental material (1 and 2).

Box 1 **Search strategy and inclusion criteria**

We performed a narrative review^33^ to find descriptions of ethical issues both in non-inferiority (NI) study protocols and in more theoretical or essayistic papers. We searched PubMed, CINAHL and EMBASE up to 12 May 2025 using a search strategy containing terms related to ‘non-inferiority’, ‘mortality’ and ‘ethics’. We included articles that had ethical issues as a main or a subsidiary subject; we excluded purely methodological or statistical papers. We included protocol descriptions because we thought these were likely to address considerations regarding the ethical issues mentioned above. Our search initially resulted in 238 studies (after removing 223 duplicates). After screening on title and abstract, 21 articles remained. After reading the full text of these 21 articles, 12 were retained in the results.^10^,^1934 35^ In this paper, we illustrate the results of the search using the PROFID-EHRA trial. Whenever necessary because of a lack of useable references, we supplement our narrative description with papers that discuss ethics and NI trials not specifically in relation to mortality.

## The choice between a superiority and a non-inferiority study

None of the protocol papers in our review reflected on the ethical underpinnings of the decision not to do a superiority but an NI study. Riechelmann *et al*, in a systematic review on NI trials in oncology, found that, in 75 trials, the most frequently mentioned reason was a benefit-harm trade-off, aiming to demonstrate that either a more convenient or a less toxic treatment was not unacceptably worse than the standard.^10^ In their review, 13 trials gave no clear reason for doing an NI study. In the more theoretical papers, NI trials are usually considered justified when they are designed to show sufficient advantage (superiority) in terms of some secondary characteristic, such as side effects or ease of use. Our example, the PROFID-EHRA trial, tries to show that a strategy without an ICD is not worse than OMT with regard to mortality, but is substantially better in terms of certain severe secondary outcomes, such as prolonged hospitalisation due to ICD lead endocarditis with sometimes lasting damage or even death due to embolisation of infected material, as well as patient burden, or costs. So here, too, the choice for an NI design is justified by a benefit-harm balance, where secondary benefits are supposed to outweigh the potential harm of an increase in mortality.

In the literature, we did not encounter the rather obvious ethical justification that would say: if a superiority design would be chosen, it would have a higher chance of failure than a more modest NI design, with the consequence that a possibly, or even probably, unnecessarily burdensome treatment would continue to be the standard. A related remarkable aspect of the protocol papers we reviewed was that usually no full-fledged statistical proof of the secondary advantages was aimed for or advocated. This is surprising, because an improvement (‘superiority’) in secondary outcomes is, as we have seen, central to the justification of these trials. After all, a study that is powered to show NI with regard to the primary outcome may not be sufficiently powered to prove superiority on secondary ones. In such cases, NI trials are underdetermined superiority studies, which in fact makes them ethically dubious. There is clearly room for improvement here.

## How to justify non-inferiority margins?

Our second question addresses acceptable inferiority. The protocol papers in our search all specified an NI margin, most often around 5% of the effect of the comparator.^11^,^17^ Only one justified the margin on clinical grounds,^15^ while the others either cited previous studies using the same margin or gave no justification. Hersh *et al* found similar figures in a systematic review.^18^ One essay argued, commenting on an NI trial in testicular cancer, for flexibility in the NI margin depending on changing clinical contexts, especially new data on the effectiveness of standard therapies.^19^ Mantovani *et al* critically discuss a cardiological NI trial that took its NI margin so wide as to allow an increase in mortality of up to 30%, in relative terms, compared with the standard treatment.^13^ They considered this unethical, although without further argumentation or proposing a generally acceptable margin, and called for major caution in performing NI trials. Hersh *et al*, in their systematic review, analysed 67 NI studies with mortality as a single or a composite end point.^18^ The average prespecified NI margin was 2.8%, ranging from 0.4% to as high as 19%. The latter number would mean that, if 100 participants were treated in both arms and the standard treatment saved 10 of them, then any figure smaller than 1.9 more deaths in the new treatment arm would be considered compatible with NI.

Our example, the PROFID-EHRA protocol, takes 1.336 as its NI margin to calculate the 95% CI and the number of patients needed to include. It justifies this with the following reasoning: “…the non-inferiority margin was taken marginally larger than the inverse of the historic HR 0.75 (for proportions) of ICD vs no ICD implantation”. While the NI margin is essentially a statistical construct used for power calculations, it can have real consequences, which need to be weighted both clinically and ethically. In the PROFID-EHRA study, depending on the real event rates in the trial, the claim of NI would be lost from a relative risk of approximately 1.11, that is, if the rates in the no-ICD arm would be 11% higher than the event rates in the ICD arm. Since previous studies have shown an annual mortality risk 4.75% in ICD carriers, this study would consider a risk below approximately 5.26% in the group without an ICD to be non-inferior compared with 4.75% in those with an ICD. So, the study would accept an absolute risk difference of around 0.5% higher risk in the no-ICD group as compatible with NI, because this would still be justified by the secondary benefits, mainly avoiding severe complications of ICD implantation.

For studies that have mortality as their primary outcome, establishing an acceptable NI margin may be harder than for other studies, because this implies considering the possibility of an increase in mortality to be irrelevant in view of the secondary benefits^9^; this is why the EMEA considered such trials ethically particularly difficult to justify.^6^ A recent article mentioned the NI margin in the PROFID-EHRA trial, noting that “transparency in statistical assumptions is essential to ensure clinical and ethical acceptability, especially when outcomes may include preventable arrhythmic death”.^20^ We fully agree with the need for transparency, and find that public justification of the NI margin in future projects might require even closer collaboration between statisticians, clinicians and ethicists to draft these explanations. However, we do not think that in principle the balancing of a chance of higher mortality (the NI margin) against substantial secondary benefits is always so difficult. It may be exaggerated, and even patronising, to say (without asking them) that people are never prepared to run a somewhat higher risk of dying if the new treatment would have a high chance of improving their lives through a decrease in side effects or other burdens and risks. After all, this is what they often are asked when it comes to risky medical procedures.

So, clearly, the important question is: who can judge whether the secondary benefits sufficiently outweigh the increased risk of harm given in the NI margin? The obvious answer, although one with substantial consequences, would be: the patient. Kim *et al*^21^ argue for involving patients in decisions on the acceptability of a trial: “Most importantly, the non-inferiority margin should reflect the level of efficacy patients are willing to forego for other benefits and not just the opinion of clinicians”. There are a few publications on how to do this. Acuna *et al* propose using systematic trade-off approaches for the incorporation of patient preferences in the NI margin,^22^ while Garfinkle *et al* report on using a Delphi procedure involving both patients and physicians to establish NI margins.^23^ We find that including the patient perspective would strengthen discussions by research ethics committees on the acceptability of NI trials.

Finally, most discussions of the NI margin are about the risk of taking it too wide (because of sample availability restrictions), potentially favouring a non-inferior outcome even when this is unjustified. But the opposite, taking the margin too small, may be equally ethically dubious, because it may make proving NI very difficult and thus, unduly favour existing therapies and interests. For instance, in the PROFID-EHRA trial, an overly narrow margin might favour ICD implantation (and the commercial interests of ICD producers) and continue avoidable patient burden. So, in conclusion, nothing absolute can be said about the NI margin; it crucially depends on the expected secondary advantages of abandoning the standard strategy and the value that physicians and patients attach to these advantages.

## What degree of certainty is justified?

To our surprise, the uncertainty inherent in any study received no attention in the literature we reviewed, even when the NI studies involved mortality. The question is how certain the results of this type of trial should be—according to the pre-established NI margin—before they can be considered credible, usually expressed through the CI. Is 95% CI sufficient, or should a 99% CI be required when mortality is the end point of an NI study? How strongly errors—both type I and type II—should be avoided is a crucial ethical question related to the value attached to different consequences—in the PROFID-EHRA trial example, either continuing to implant unnecessary ICDs or withholding ICDs. One might argue that an NI trial may need stronger guarantees of certainty than a superiority trial, because its consequence may lead to the abandonment of long-standing and strongly entrenched clinical strategies. Moreover, it may be harder to adequately inform participants about how to weigh the trade-off between a less invasive strategy that may—or may not be—less safe and effective. On the other hand, demanding a higher degree of certainty may have similar drawbacks as very narrow NI limits, such as the need for a higher number of study participants, and the increased difficulty of demonstrating NI when it is there. In other words, demanding a very high degree of certainty might facilitate a bias towards conservatism. We think that, in the end, there is no justification for stricter certainty requirements in NI trials, because the arguments for and against such stricter requirements are equally strong.

## How should participants be informed?

In any trial, participants need to be informed properly to be able to decide whether they want to participate or not. The consequences of both participation and non-participation should be clearly described. Against this background, questions may be raised about informed consent in NI trials with mortality as a primary end point. Maybe the most important one is: what do participants need or want to know about the exact purpose of an NI trial and is this different from superiority trials or other study designs? In a study by Doshi *et al*, participants and methodologists assessed information leaflets of 50 NI trials studying antibiotics.^24^ They found that the study purpose was explained in only 11 of these according to participants, and in just one according to methodologists. They regarded this as unacceptably low. Moreover, none of the trials had an explicit clinical justification for the NI margin that was chosen—let alone that participants were informed about it.

In an editorial accompanying the study, Menikoff argued that these results are less alarming than the authors thought, for several reasons.^25^ First, the purpose of NI studies is more complicated to explain than that of superiority studies; second, full explanation may lead to the idea that the index treatment is inferior, or that the importance of study is too marginal to want to participate, and third, participants have reasons to be more interested in information about risks and burdens of study participation than in the exact aims of the study. In fact, what Menikoff suggests in the second point could be restated as a form of the so-called therapeutic misconception, which in superiority trials is supposed to lead to a bias towards trial participation by patients hoping to receive the experimental treatment. In the case of NI studies, however, it would likely lead to non-participation by patients who fear ending up in the experimental arm; but in both cases, avoiding misconceptions demands better explanation of the study, not refraining from explanation altogether. Menikoff’s view is clearly contrary to the Helsinki Declaration, which demands informing participants about the aim of the study. He seems to think that NI studies are different in this respect from superiority studies, but it is unclear why that would be the case. The review by Doshi *et al* was not about studies with mortality as end points, but their worries might be even stronger when mortality is at stake because, in such studies, participants may be more than usually interested in the aims and the potential consequences of the study.

Even if explaining the aim of an NI trial with mortality end points to participants is important, it is likely to be more difficult than for superiority trials. Jatoi and Gail give an example of how this difference may work out.^26^ For a superiority trial, they say the explanation may be something like this: “We do not know if the new treatment is better than the standard treatment, but we hope it is, and this trial is designed to see if it is better”. For NI trials, they propose a formula that they describe as true but awkward: “We do not know if the new treatment is better or worse than the standard treatment, but we want to establish that if it is worse, it is only a little worse than the standard treatment”. Less awkward formulas may need to be developed, but this attempt shows that it is not impossible to explain the aim of NI studies to participants. We think a less awkward explanation might say something like this: “The treatment we currently use for your condition is effective, but has disadvantages. We are now testing whether a different treatment does not have these disadvantages and is no more than X percent less effective than the current treatment”. However, potential participants should be the ones to say which formulation is most appropriate. Within the PROFID project, we are currently conducting a survey addressing, among others, participants’ consent preferences including what information people want about the aims of an NI trial.

## Discussion

We conclude that regarding NI studies with mortality as an end point, the questions we raised, and the answers we found, need to be taken into account by researchers and ethics committees. On the basis of our findings, we tried to formulate some preliminary guidance on these issues. Mainly, a higher mortality risk can in principle be balanced by a decrease of secondary burdens or risks—patients do this often in clinical care. So the EMEA statement we quoted,^6^ about the great difficulty of establishing an NI margin for mortality NI studies should not be taken to mean that this is impossible. Even though some degree of increased risk may be considered impermissible by everyone, there is no standard numerical value of acceptability and in our view there cannot be one. We noted that NI trials are more appropriately called ‘acceptable-inferiority trials’, because there is always some level of risk to be accepted. While in this article we continued using the term ‘NI trial’ for familiarity and readability purposes, hereafter we reflect on the important issue of acceptability.

The acceptability can only be determined on a case-by-case basis, involving, among others, research ethics committees. In fact, the need for independent assessment of the harm-benefit ratio is the reason why ethics review of every clinical trial is necessary. A comparison with similar risks that are considered acceptable or unacceptable is often helpful for this. That being said, we find that this balancing also demands, first, that patients, or their representatives, are involved in discussions about the NI margin. In the end, only patients from the population under study can say whether the decrease of burden and secondary effects justifies an increase in mortality risk. Even though we found two studies explaining how patients could be involved, this is largely uncharted territory. It also demands, second, that the aim of an NI trial is clearly explained to potential participants: the NI margin should be explained in the participant information and discussed with individual potential participants.

It is likely that the views of participants on the acceptability of NI margins will depend on the expected secondary benefits; if participants consider these not very important or relevant, the margin will need to be closer to zero than if they find them really critical. In such cases, NI studies may also need stricter uncertainty limits (ie, small CIs). It is not clear, however, whether the advantages of choosing a higher level of certainty than 95% outweigh the disadvantages—such as the increased number of participants required. Since the arguments for and against stricter certainty requirements in NI trials are equally strong, we find no clear justification for mandating narrower CIs.

In the context of determining acceptability, the framing of the NI margin is crucial. As Tversky and Kahneman have shown >40 years ago, different framings of the same numerical data can lead to diametrically opposed judgements about acceptability.^27^ This is also shown by more recent survey studies analysing understandings of relative risk and absolute risk: in 1993, Malenka *et al* found that 60% of patients opted for a medication when benefit was framed in relative terms, compared with only 15% when benefit was given in absolute terms.^28^ More recently, Perneger and Agoritsas performed a clinical trial with different framings of the effects of a treatment and they found that “describing clinical trial results as absolute risks is the least biased format, for both doctors and patients”, and that perception of the absolute risk format was the same as for a ‘fully informed’ condition where both absolute and relative risks were given.^29^ However, both in communication towards patients and in medical journals, the benefits are generally described only in relative terms—making the difference seem larger—while harms are presented in absolute terms to make them appear smaller. Thus, clinicians often misinterpret these statistics, leading to excessive enthusiasm about benefits of superiority trials.^30^ Conversely, for NI trials, reporting the risk in relative terms could lead to excessive worry. As mentioned above, the PROFID trial would consider an absolute mortality risk difference of 0.5% (5.26 instead of 4.75%) acceptable. This difference can also be expressed as a relative increase of 11%. We believe that explaining the absolute increase in risk related to the trial is generally preferable to explaining only the relative risk increase, as the latter does not convey an individual’s actual risk and can be misleading—potentially exaggerating a small increase by making it seem large. This biased perception could also hinder the conduct of the NI trial, as excessive concerns about risks may arise when they are framed as being very large.

Also, one of the challenges ahead is the need to rethink the distinction between primary and secondary outcomes. As mentioned above, NI studies can be seen as superiority studies with composite outcomes that include what are currently considered secondary end points. While this topic is beyond the scope of this paper, we note that using composite end points in trials is generally challenging, and study designs should avoid combining outcomes that are expected to move in opposite directions.^31^ Going back to the example of PROFID-EHRA, we think that the potential secondary benefits of abandoning ICD implantation in selected patients are large enough to justify this mortality end point NI trial. However, because it is such an ethically difficult area, a parallel ethical analysis is part of the PROFID project with the double aim of strengthening the trial’s ethical justification and of learning lessons for other NI trials with mortality as an end point. Since patient views are crucial for the justification of the NI studies, these views will be studied as part of a larger patient survey within the PROFID project.

Our line of thinking may also be relevant for studies that have mortality as a secondary end point. Such secondary end points are sometimes not communicated to participants (eg, the SUPPORT trial^32^), because the researchers do not expect a mortality difference, or maybe hope there is none, or find it too difficult to explain. We think our analysis shows that uncertainties about mortality effects can be and should be discussed with participants, even if these are not the primary end point of the study.

For now, we conclude that NI trials of life-saving treatments can be justifiable because, in general, patients may be prepared to trade a larger mortality risk against a chance of better quality of life or other benefits. However, we do argue for explaining to potential participants the trade-off between a higher mortality risk against substantial secondary benefits. This trade-off will likely be different for different participants.

## Supplementary material

10.1136/jme-2024-110517 online supplemental file 1

## Data Availability

No data are available.

## References

[1] Leung JT, Barnes SL, Lo ST (2020). Non-inferiority trials in cardiology: what clinicians need to know. Heart.

[2] Mauri L, D’Agostino RB (2017). Challenges in the Design and Interpretation of Noninferiority Trials. N Engl J Med.

[3] van Buchem FL, Knottnerus JA, Schrijnemaekers VJ (1997). Primary-care-based randomised placebo-controlled trial of antibiotic treatment in acute maxillary sinusitis. Lancet.

[4] Garattini S, Bertele’ V (2007). Non-inferiority trials are unethical because they disregard patients’ interests. Lancet.

[5] Nunn AJ, Meredith SK, Spigelman MK (2008). The ethics of non-inferiority trials. Lancet.

[6] EMEA (2005). Guideline on the choice of the non-inferiority margin. Doc. Ref. EMEA/CPMP/EWP/2158/99. https://www.ema.europa.eu/en/documents/scientific-guideline/guideline-choice-non-inferiority-margin_en.pdf.

[7] Dagres N, Peek N, Leclercq C (2020). The PROFID project. Eur Heart J.

[8] Fleming TR, Odem-Davis K, Rothmann MD (2011). Some essential considerations in the design and conduct of non-inferiority trials. Clin Trials.

[9] Annoni M, Sanchini V, Nardini Cecilia (2013). The ethics of non-inferiority trials: A consequentialist analysis. Res Ethics.

[10] Riechelmann RP, Alex A, Cruz L (2013). Non-inferiority cancer clinical trials: scope and purposes underlying their design. Ann Oncol.

[11] Bonnet F, Bénard A, Poulizac P (2020). Discontinuing statins or not in the elderly? Study protocol for a randomized controlled trial. Trials.

[12] Hayakawa M, Tagami T, IIjima H (2020). Restrictive transfusion strategy for critically injured patients (RESTRIC) trial: a study protocol for a cluster-randomised, crossover non-inferiority trial. BMJ Open.

[13] Mantovani V, Lepore V, Mira A (2010). Non-inferiority randomized trials, an issue between science and ethics: the case of the SYNTAX study. Scand Cardiovasc J.

[14] Bitterman R, Koppel F, Mussini C (2021). Piperacillin-tazobactam versus meropenem for treatment of bloodstream infections caused by third-generation cephalosporin-resistant Enterobacteriaceae: a study protocol for a non-inferiority open-label randomised controlled trial (PeterPen). BMJ Open.

[15] Coutsoudis A, Daniels B, Moodley-Govender E (2016). Randomised controlled trial testing the effect of cotrimoxazole prophylaxis on morbidity and mortality outcomes in breastfed HIV-exposed uninfected infants: study protocol. BMJ Open.

[16] Li C, Ni Y, Liu C (2023). Mediastinal lymph node dissection versus spared mediastinal lymph node dissection in stage IA non-small cell lung cancer presented as ground glass nodules: study protocol of a phase III, randomised, multicentre trial (MELDSIG) in China. BMJ Open.

[17] van der Zaag AY, Bhagirath SC, Boerman AW (2024). Appropriate use of blood cultures in the emergency department through machine learning (ABC): study protocol for a randomised controlled non-inferiority trial. BMJ Open.

[18] Hersh AM, Walter RJ, Abberegg SK (2019). Use of Mortality as an Endpoint in Noninferiority Trials May Lead to Ethically Problematic Conclusions. J Gen Intern Med.

[19] Cullen MH, Stenning S (2004). Clinical trials with moving targets: a commentary on a non-inferiority trial in testicular cancer. Lancet Oncol.

[20] Fauchier L, Bodin A, Pierre B (2025). High mortality after ST-segment elevation myocardial infarction and implications for implantable cardioverter defibrillator trials design. Europace.

[21] Kim KS, Chan AW, Belley-Côté EP (2022). Noninferiority Randomized Controlled Trials. J Invest Dermatol.

[22] Acuna SA, Chesney TR, Baxter NN (2019). Incorporating Patient Preferences in Noninferiority Trials. JAMA.

[23] Garfinkle R, Sabboobeh S, Demian M (2021). Patient and Physician Preferences for Antibiotics in Acute Uncomplicated Diverticulitis: A Delphi Consensus Process to Generate Noninferiority Margins. Dis Colon Rectum.

[24] Doshi P, Hur P, Jones M (2017). Informed Consent to Study Purpose in Randomized Clinical Trials of Antibiotics, 1991 Through 2011. JAMA Intern Med.

[25] Menikoff J (2017). What Should Patients Be Told About Noninferiority Studies?. JAMA Intern Med.

[26] Jatoi I, Gail MH (2020). The Need for Combined Assessment of Multiple Outcomes in Noninferiority Trials in Oncology. JAMA Oncol.

[27] Tversky A, Kahneman D (1981). The framing of decisions and the psychology of choice. Science.

[28] Malenka DJ, Baron JA, Johansen S (1993). The framing effect of relative and absolute risk. J Gen Intern Med.

[29] Perneger TV, Agoritsas T (2011). Doctors and patients’ susceptibility to framing bias: a randomized trial. J Gen Intern Med.

[30] Caverly TJ, Prochazka AV, Binswanger IA (2014). Confusing Relative Risk with Absolute Risk Is Associated with More Enthusiastic Beliefs about the Value of Cancer Screening. Med Decis Making.

[31] Tomlinson G, Detsky AS (2010). Composite end points in randomized trials: there is no free lunch. JAMA.

[32] SUPPORT Study Group of the Eunice Kennedy Shriver NICHD Neonatal Research Network (2010). Target Ranges of Oxygen Saturation in Extremely Preterm Infants. N Engl J Med.

[33] Sukhera J (2022). Narrative Reviews: Flexible, Rigorous, and Practical. J Grad Med Educ.

[34] Muller G, Kamel T, Contou D (2021). Early ve**r**sus differed arterial catheterisation in critically ill patients with acute circulatory failure: a multicentre, open-label, pragmatic, randomised, non-inferiority controlled trial: the EVERDAC protocol. BMJ Open.

[35] Rothmann M, Li N, Chen G (2003). Design and analysis of non-inferiority mortality trials in oncology. Stat Med.

