# Supplementary material for: How can non-inferiority studies with mortality end points be ethically justified?
Source: J Med Ethics. 2025 Aug 26;52(6):e110517. doi: 10.1136/jme-2024-110517 (PMC13311967; doi:10.1136/jme-2024-110517)

Appendix 1: Search strings

Studies included up to September 3, 2024

Pubmed

# Noninferiority	"noninferiority"[tiab] OR "non-inferiority"[tiab]	15,302
# Mortality	"mortality"[tiab] OR "survival"[tiab] OR "mortality"[MeSH]	2,309,445
# Ethics	"ethic*"[tiab] OR "ethics"[MeSH] OR "consent"[tiab]	317,000
Combined	#1 AND #2 AND #3	125

Embase

# Noninferiority	noninferiority.ti,ab OR non-inferiority.ti,ab	24680
# Mortality	mortality.ti,ab OR survival.ti,ab OR exp mortality/	3556509
# Ethics	ethic*.ti,ab OR exp ethics/ OR consent.ti,ab	574494
Combined	#1 AND #2 AND #3	301

CINAHL

# Noninferiority	(TI (noninferiority OR non-inferiority)) OR (AB (noninferiority OR non-inferiority))	5,761
# Mortality	(TI (mortality OR survival)) OR (AB (mortality OR survival)) OR (MH (“Mortality+”))	446,560
# Ethics	(TI (ethic* OR consent)) OR (AB (ethic* OR consent)) OR (MH (“Ethics+”))	208,272
Combined	#1 AND #2 AND #3	35

Appendix 2: PRISMA flow chart

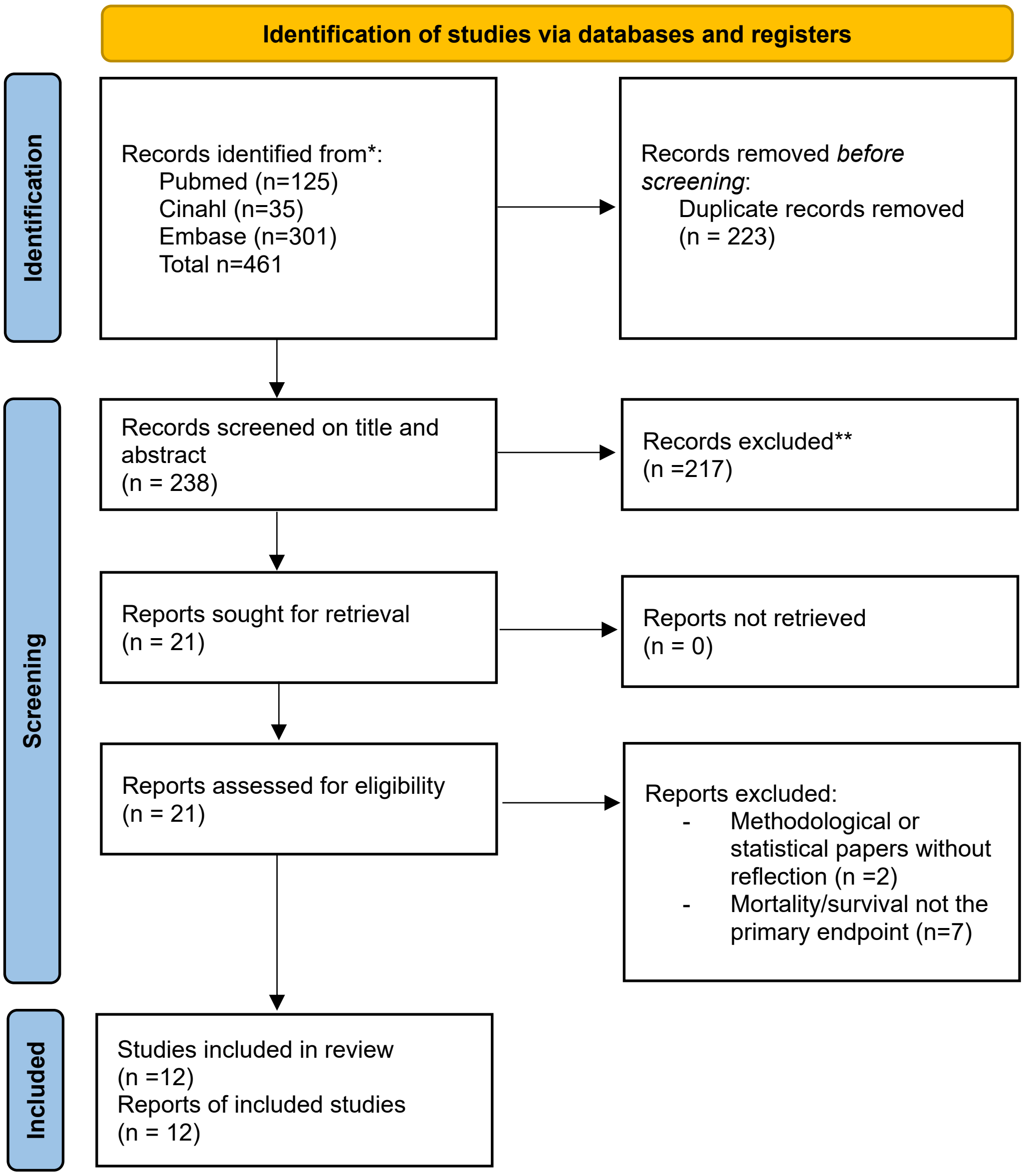

Supplement: online supplemental file 1 [file jme-52-6-s001.pdf]
